# Situational Judgement Tests among Palestinian community members and Red Crescent volunteers to inform humanitarian action: a cross-sectional study

**DOI:** 10.1186/s13690-024-01356-8

**Published:** 2024-08-27

**Authors:** L. S. Moussaoui, M. Quimby, H. Avancini, A. Salawdi, F. Skaik, R. Bani Odeh, O. Desrichard, N. Claxton

**Affiliations:** 1 Faculty of Psychology and Educational Sciences, Geneva University, Geneva, Switzerland; 2 Nadulpan LLC, Crestview, FL USA; 3 Swedish Red Cross Society, Stockholm, Sweden; 4 Palestine Red Crescent Society, al-Bireh (Ramallah and al-Bireh), Palestine

**Keywords:** Situational judgment tests, Social norms, Waste mismanagement, Violence, Road accident

## Abstract

**Background:**

Informing humanitarian action directly from community members is recognized as critical. However, collecting community insights is also a challenge in practice. This paper reports data collected among community members and Red Crescent volunteers in the occupied Palestinian territory. The aim was to test a data collection tool, situational judgment tests (SJTs), to collect insights in the community around three themes.

**Methods:**

The SJTs covered violence prevention, road safety, and environmental pollution (waste), and were constituted of hypothetical scenarios to which respondents indicated how they would react. For each theme, the answers’ pattern provides insights for humanitarian action regarding which beliefs to address. A cross-sectional survey was conducted in January and February 2023 with 656 community members, and 239 Red Crescent volunteers.

**Results:**

Data showed that violence is the theme for which the need is the highest among community members. Some responses varied according to the public (age, governorate, or disability level), suggesting actions could be tailored accordingly.

**Conclusions:**

Despite many difficulties during data collection, this study show that the tool allowed to collect community insights, a crucial task to ensure adequate response to the challenges faced by community members and Red Crescent volunteers in occupied Palestine.

**Supplementary Information:**

The online version contains supplementary material available at 10.1186/s13690-024-01356-8.

**Table:** 

Text box 1. Contributions to the literature
• Self-report survey is the most frequently used method to measure beliefs and perceptions among community members
• Self-report survey limitations are recognized but there are limited alternatives to collect data in the field
• This paper reports the test of a method, situational judgements tests, originally used in occupational psychology (employees’ selection, school admissions) as an alternative to measure beliefs and norms
• Situational judgements tests allowed to collect data in Palestinian communities about three different topics (violence, road safety, waste management)

## Background

Local actors are crucial informers of the needs of a community, as was formally recognized at the World Humanitarian Summit in 2016 [1]. The focus on community engagement indicates that the communities being served are a critical part of the team that conducts the assessment, planning, implementation, and evaluation of any intervention plan which exceeds the general expectation that “services are responsive to community needs and inputs” [2]. Minimum quality standards and indicators for community engagement [3] ensure, among other things, that communities are meaningful stakeholders in ongoing two-way communication and that programs are aligned with local needs and priorities and are decided with and by community members.

Over 154 Red Cross and Red Crescent National Societies rely on this strategy for the delivery of programs using a Community-Based Health and First Aid (CBHFA) approach with volunteers from the communities leading the identification of challenges, issues and barriers as well as leading the solving of any issues with and by community. Few studies have examined the community-based approach, especially in conflict-affected contexts [4]. Moreover, a lack of standardization in data collection for community engagement has been highlighted [2]. This study aims to report the use of Situational Judgement Tests as a method to collect community insights in three specific areas from Palestinian community and Palestine Red Crescent volunteers working in select areas of the occupied West Bank.

### Advantages of community insights

In occupied Palestinian territory, local actors such as Red Crescent volunteers serve in the very communities where they live, work and play. This community-level access brings many advantages for preparedness for crises as well as in humanitarian response: community health workers and volunteers have a deep understanding of the unique context and the changing needs [5, 6], and, unlike international responders, they are a constant presence before, during and well after a crisis [4]. They overwhelmingly contribute to ongoing access and support in the long term [7]; they know the unique needs of their community, speak local languages and dialects; and, they understand the culture to know what is feasible and what is not changeable. *Community-based volunteers* designate people who experience the conflict and suffer its consequences [4] while also providing support to others in the same situation. In this paper, local actors/community-based volunteers encompass community members and Red Crescent volunteers.

Some rare papers report examples of the use of community insights to guide organizational responses. Most of them are in the field of emergency response to epidemic response. One example is Bedson et al. [8], who used quantitative (epidemiological) and qualitative data to develop *Community-led Ebola Action approach*. Qualitative data measured the most commonly expressed concerns, the perceived risks of contracting Ebola, and action plans developed in the community. Data was used to establish feedback loops between communities and authorities, and inform response services.

Baggio [9] presents how real-time data was used during the Ebola outbreak in the Democratic Republic of Congo. Red Cross volunteers measured the perceptions and needs of affected communities thrice weekly to tailor the response that aid organizations could provide. Data was collected using a simple form recording comments, which were then entered in a Microsoft Excel log sheet. The analysis of the content provided adapted communication and programs following local trends, for example, to address misconceptions.

Building upon the experience with Ebola, Erlach et al. [10] report how community feedback was used to guide Red Cross and Red Crescent response against COVID-19 in Sub-Saharan Africa. Similarly, content emerging from interactions with community members (such as questions, beliefs, and rumors) was collected and translated into priority responses.

Colombo and Pavignani [11] analyzed key failings in humanitarian health action. Among others, one that is especially relevant for this paper is the poor communication between humanitarian workers and those they are meant to serve (or between distant managers and frontline workers), which creates distance and inadequate response, because it is not contextualized nor tailored to local needs. Colombo and Checchi [12] further argue that a culture of immediacy in the response provision, deriving from addressing basic needs at the beginning of an acute crisis, might lead to a tendency to act without a proper situation analysis.

In the cases where, as in CBHFA programming, local residents are affiliated with the organization providing support, the distance mentioned by Colombo and Pavignani [11] disappears. However, using data to inform action advocated by Colombo and Checchi [11] is not straightforward even if locals/community-based volunteers constitute the team that will provide a program. Volunteers might provide their own perception of the priorities, but they don’t necessarily have access to the beliefs held by all community members, and are not necessarily representative of the general population in terms of demographics. For example, in the occupied Palestinian territories, a majority of volunteers are women, because CBHFA was originally formed as mothers’ clubs and the population still see CBHFA as female-focused. Thus, collecting data directly in the communities while necessary, comes with difficulties.

### Difficulties associated with community insights

One difficulty evoked in the paper by Erlach et al. [10] on the system set-up to collect community insights on COVID-19 perceptions is the impossibility of matching the collected inputs to sociodemographic variables (because of the way feedback was collected), preventing a finer analysis from being conducted. The authors advocate for triangulation with structured surveys. In the same vein, Bedson et al. [2] evoke that there has been limited standardization in data collection as part of community engagement and argue for adoption of standardized measurements.

### SJT as a tool to collect data from the community and the volunteers to guide programs

Some limitations are associated with the use of structured surveys. Notably, Likert scales are widely used to measure respondents’ opinions and beliefs but have been evaluated as not optimal [13–15]. The format (order of the response options and direction of the question) influences answers [13, 14]. And cultural differences have been highlighted in terms of answer style or difficulty in choosing one option [15–18]. In a study in Sierra Leone, Moussaoui et al. [19] used Situational Judgment Tests (SJTs), most of the time used for school admissions and employees’ selection, to measure beliefs and norms of the community about several topics of sexual and reproductive health. SJTs are hypothetical scenarios in which the interviewee is asked to select their typical response among several possibilities. In the context of job and school selection, meta-analyses have demonstrated that they are reliable and valid predictors of performance [20]. Meta-analytical data also supported the tool’s satisfactory test-retest reliability [21]. Compared to other assessment methods, they are convenient for large-scale delivery and cost-efficient once developed [22]. According to Lipnevich et al. [23], SJTs are less subject to bias frequently present in self-reports, notably trying to guess which answer the interviewer expects. SJTs are written to make it difficult to give what people may feel is the ‘right’ answer as the tests pose situational prompts that the interviewee encounters or may encounter in their lives within a specific context. Situational judgment test items consist of two elements: the scenario that gives the situation to be solved and the possible actions from which a person can choose.

In a previous study in Sierra Leone [19], data showed that SJTs answers had positive but of moderate magnitude correlations with self-report items on the same topic. For example, answers to an SJT about one’s hypothetical reaction if their partner slaps them correlated with the knowledge of action to take if violence is witnessed. Among respondents who did not know what immediate action to take if they were witnesses of violence, none chose the highest-scored SJT answer (telling the person they are no longer together). On the contrary, among persons who knew three or more actions to take if they were witnesses of violence, none of them answered the lowest-scored SJT answer (put up with the abuse and hope it gets better). Although preliminary, those results suggest SJT might be an interesting tool to measure stigma-sensitive norms.

Identifying a reliable tool to measure beliefs in communities is essential because if the answers are biased (when respondents are trying to provide the “correct” answer), then the situation analysis will be biased too, and the resulting program will likely miss its point. Aside from reliability, authors have argued for developing easy-to-understand and acceptable (in terms of topic sensitivity) questionnaires [12]. SJTs have the advantage of presenting daily life situations with a range of plausible responses. Thus, it should be easier for respondents to understand the question than a more abstract way of asking questions.

### Focus on the Palestinian context and the three themes studied

We used three SJTs within a broader existing survey to measure norms and beliefs of a select group of villages served by Palestine Red Crescent volunteers. The three questions posed focused on three topics identified in recent Vulnerability and Capacity Assessments which were considered local priorities. The three SJTs consisted of one question each on violence, road accidents, and waste management.

The first topic covered in this study is violence. In the context of the Israeli-Palestinian conflict, political violence has been present for decades [24]. It is noted that the unique context of occupied Palestine by Israel posed considerably higher levels of stress than in other complex situations. However, the issue of violence is not restricted to political violence only, as studies showed that exposure to political violence is associated with other types of violence, such as family violence [25] and intimate-partner violence [26]. A study published in 2020 showed that half of a sample of young Palestinians had personally been victims of violence, and more than two-thirds witnessed or heard about violence perpetrated on a close one [27]. As stated in a WHO report [28], “deaths and injuries are only a fraction of the burden” (p.8). Studies have shown that violence exposure has consequences for the mental health of individuals. Notably, Wagner et al. showed that young Palestinians more exposed to violence had higher rates of global distress, depression and anxiety, and the effect was stronger for females [27]. The SJT we posed in the survey referenced staying safe from violence in general.

Around the world, the number of road traffic crashes and related deaths is extremely high [29]. Road accident causes injuries, deaths, and are also an economic burden for the country [30]. In occupied Palestinian territories, data show an increasing trend in accidents between 1971 and 2001 [31] and, more recently, between 1994 and 2015 [32]. Differences have been noted according to regions (e.g., West Bank compared to Gaza Strip) explained by the difference in population density and motorization rates [33]. The authors analyzed that 75.8% of accidents are caused by drivers’ lack of adherence to traffic law and improper driving [33].

Waste mismanagement is another issue preponderant in occupied Palestinian territories but also worldwide. A report from the World Bank cites a (conservative) estimation of at least 33% of waste being mismanaged through open dumping or burning [34]. Ferronato and Torretta [35] review the environmental impacts of open dumping and open burning and cite marine litter, air, soil and water contamination, and the health impacts due to exposure to hazardous waste as the main issues. The lack of sanitary landfills in occupied Palestinian territories leads to dumping and open burning [36]. A study based on a national household sample survey showed that organic waste accounts for more than 81% of residential solid waste [37]. In the occupied West Bank, it has been estimated that there are 0.034 mechanical treatment facilities per 100’000 inhabitants and zero composting facilities for the same number of inhabitants [38]. Thus, the authors suggest that home composting could be an effective approach for this waste fraction.

### Research question

Can SJTs be used to measure the norms and beliefs around violence, road safety, and environmental pollution? And do this method allows to detect variations in answers according to socio-demographic groups and geographical regions?

## Methods

Both qualitative and quantitative data collection methods were employed in the broader survey, including secondary data review, a community member survey, a volunteer survey, focus group discussions, and key informant interviews. As part of the community and volunteer surveys, three SJT questions were inserted in each survey, addressing three of the top public health risks. These SJTs were included within the community and volunteer surveys in an attempt to measure norms and attitudes around some specific health behaviors.

### Sampling strategy

The sampling strategy was based on population data (male and female above 18 years old) and volunteer numbers in each governorate (Hebron, Bethlehem, and Central/Jerusalem). The team aimed for a disproportional stratified random sample, which meant that we wanted approximately an equivalent number of respondents for each group to be able to compare them. The objective was to secure at least 68 community members respondents of each gender in each of the three governorates, leading to a planned minimum of 408 respondents.

Palestine Red Crescent Society (PRCS) volunteers served as enumerators for both surveys (Community survey and Volunteer survey). The enumerators were trained in a face-to-face session in Ramallah with the sampling strategy clearly laid out. However, due to travel restrictions from events related to the ongoing conflict (data collection took place during January and February 2023), the intended strategy was not possible. In following up with enumerators and their leads, it was determined that it was only safe to conduct the surveys via phone. Additionally, the volunteers resorted to a snowball method to gather respondents – calling people that they knew and asking for additional contacts within the three governates. It was shared that people did not answer the phone if the phone number was unknown, which was a limitation.

In the end, a total of 656 community members answered to at least one of the three SJT questions across the three governorates. 64.6% of the respondents are female and 35.4% are male. The youngest community member who responded is 19 years of age, while the oldest is 97 years of age (mean age = 41.2).

239 volunteers responded to at least one of the three SJT questions on the volunteer survey. 79.5% of responding volunteers are female, while 20.5% are male. The youngest responding volunteer is 19 years of age and the oldest is 80 years of age (mean age = 33.8).

### Material

The community and volunteer surveys, inclusive of the suggested SJTs were prepared, discussed, and edited between the lead researchers, Swedish Red Cross, and Palestine Red Crescent. They were then translated by Palestine Red Crescent. There was not a secondary review of the translations to ensure accuracy of the questions and responses.

Just as real-world situations are never entirely black or white, SJT scenarios sometimes do not have just one right or wrong answer. The response options of an SJT item can contain one action that is the most appropriate for the question asked in that situation (which earns full points), one or two actions that are somewhat appropriate (and earn partial points), and one or two actions that would be inappropriate for the question asked in that situation (earning no points). The answer options available are clear *actions* to take (or ways to behave) rather than results of actions. Each action is intended to be logically possible for the specific scenario (even wrong ones).

Table 1 provides the detailed content of the SJTs used in the study for the three topics, in community and volunteers’ versions.

**Table 1:** Description of the situational judgement tests and their response options, January-February 2023, Westbank region, occupied Palestinian territories

Violence SJT						
	**There is considerable risk of violence in your village. The community is constantly on high alert and there have been regular small-gunfire incidents that affect the psychosocial state and threaten people’s lives every day. What do you do to manage this stress?**					
Community	You try to stay safe but do not take any exaggerated precautions – you trust that you can trust your intuition and stay safe (4) AND You do not take any special precautions – you trust that you can trust your intuition and stay safe (4)	Work with your PRCS CBHFA volunteer to learn about violence prevention and how to provide and receive psychological first aid (3)	You regularly attend meetings in your area to understand the risks facing your family. You keep informed but try to not become paralysed by fear. (2)			You stay home as much as possible, fearful that your safety is always at risk. You also keep your family home as much as possible. (1)
Volunteers	You try to stay safe but do not take any exaggerated precautions – you trust that you can trust your intuition and stay safe (4) AND You do not take any special precautions – you trust that you can trust your intuition and stay safe (4)	Work with your PRCS CBHFA volunteer to learn about violence prevention and how to provide and receive psychological first aid (3)	You regularly attend meetings in your area to understand the risks facing your family. You keep informed but try to not become paralysed by fear. (2)			You stay home as much as possible, fearful that your safety is always at risk. You also keep your family home as much as possible. (1)
*Road safety SJT*						
	**You are fearful that your teenage sons will become involved in one of the traffic accidents**					
Community	You teach them the correct practices (wearing seatbelt, respect the speed limits) and make them practice safe driving with a licensed careful driver (5)	You work with your PRCS CBHFA volunteer to lead youth sessions about road safety and how people can stay safe in traffic (4)	You allow them to drive the family car only when you or your brother are with them (3)	You forbid your sons from driving or being in a car with their friends (2)		You tell your sons that they should not drive like you do – that you drive the way that you do because you have many years of practice and it is safe to do so (1)
Volunteers	You teach them the correct practices (wearing seatbelt, respect the speed limits) and make them practice safe driving with a licensed careful driver (5)	You work with your PRCS branch supervisor to lead youth sessions about road safety and how people can stay safe in traffic (4)	You allow them to drive the family car only when you or your brother are with them (3)	You forbid your sons from driving or being in a car with their friends (2)		You tell them every day that they must be safe – they must wear a seatbelt, drive the speed limit, and they must drive safely or you will be very disappointed (1)^a^
*Waste SJT*						
	**Your household produces a lot of rubbish with food waste**, **packaging materials**, **and other items that take up a lot of space. There is not enough space in the rubbish bin and the garbage collectors don’t come as they should. You are afraid of rats and other vermin. What do you do?**					
Community	You throw the compostable waste into a bin for your garden or to give to a friend with a garden. And you talk with your household members about ways to reduce other types of waste (5)	You discuss with your PRCS CBHFA volunteer about possibilities to reduce the waste problem at your home and in the community, for example by creating a common compost to provide nutrients to plants (4)	You watch out for vermin in the rubbish stacked up (e.g. by cleaning the place regularly and use repulsive) until the garbage collectors do come (3)	You give small bags of trash to different household members to get rid of each week wherever they can. You do not ask where they dump the trash – you are just happy it is gone. OR You take the trash to dump in the river or in a common dumping area where others you know of dump the trash (2)		You simply stack up the rubbish until the garbage collectors do come (1)
Volunteers	You throw the compostable waste into a bin for your garden or to give to a friend with a garden. And you talk with your household members about ways to reduce other types of waste (5)	You discuss with your PRCS CBHFA volunteer about possibilities to reduce the waste problem at your home and in the community, for example by creating a common compost to provide nutriments to plants (4)	You watch out for vermin in the rubbish stacked up (e.g. by cleaning the place regularly and use repulsive) until the garbage collectors do come (3)	You give small bags of trash to different household members to get rid of each week wherever they can. You do not ask where they dump the trash – you are just happy it is gone. OR You take the trash to dump in the river or in a common dumping area where others you know of dump the trash (2)		You simply stack up the rubbish until the garbage collectors do come (1)

*Note* SJT = Situational Judgement Tests, PRCS CBHFA = Palestine Red Crescent Society Community-Based Health and First Aid

^a^One response option differed between community and volunteer’s versions of the survey. This difference was due to a human error in coding the survey onto the platform, by confusing multiple versions of the survey (iterations)

## Results

### Violence SJT – community

In the community sample answering the violence SJT (*N* = 418^2^), 43.1% of respondents chose the option “Stay at home as much as possible”, 21.3% answered “Regularly attend meetings to understand risks”, 18.4% “Work with PRCS CBHFA volunteer to learn about violence prevention”, and 17.2% “Try to stay safe but do not take any exaggerated precautions” (the best answer according to our coding and agreement with PRCS).

**Fig. 1:**
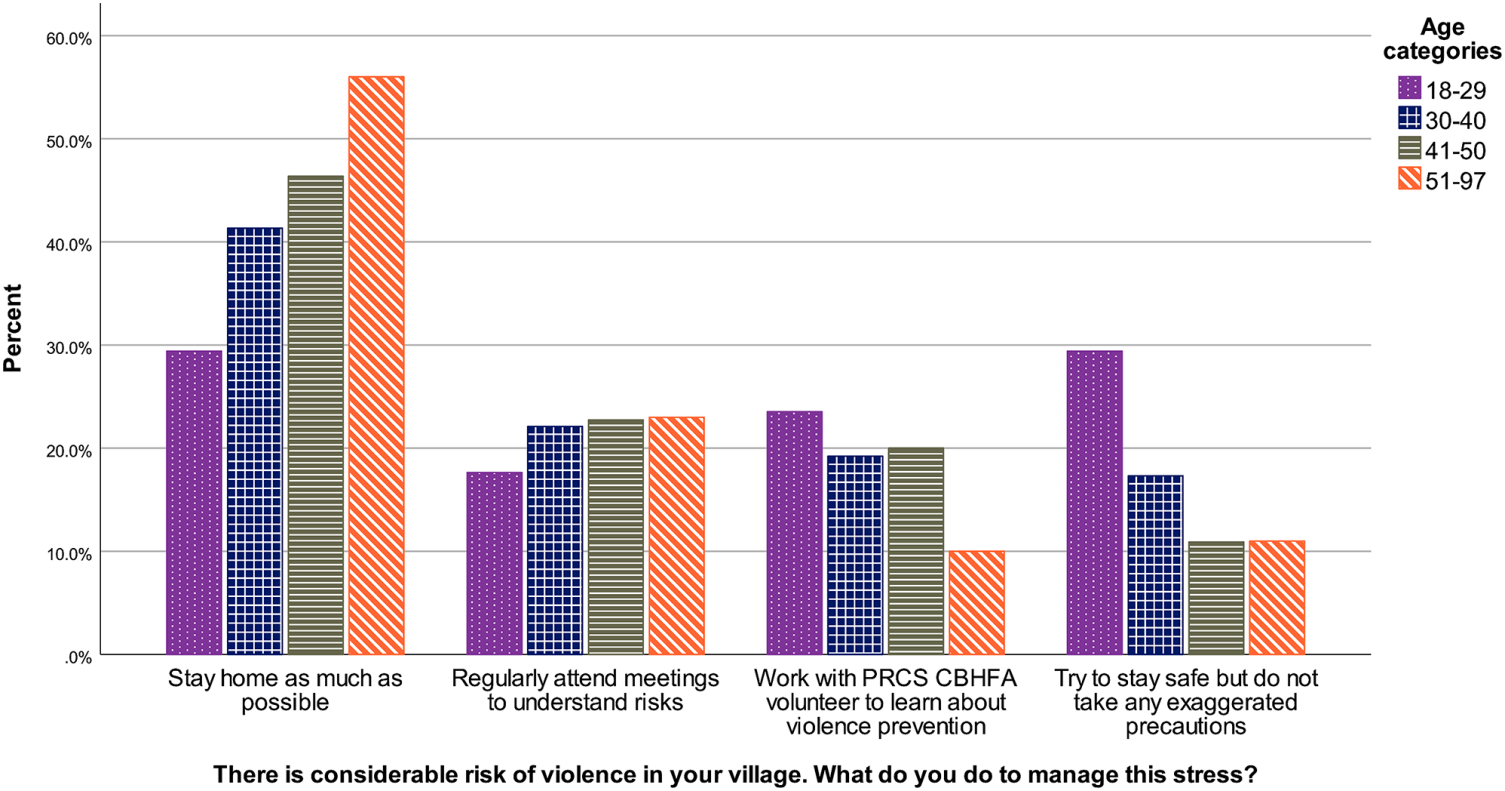
Violence situational judgement test answers according to age in the community sample, January-February 2023, Westbank region, occupied Palestinian territories *Note*. PRCS CBHFA = Palestine Red Crescent Society Community-Based Health and First Aid

There were differences in answer patterns according to the age of respondents, and Kruskal-Wallis test shows the difference is significant, *H* (3) = 24.02, *p* < .001. Results are presented in Fig. 1. It is apparent that answers from the oldest groups are more frequently “stay at home”, compared to youngest who, comparatively, try more to “stay safe without taking any exaggerated precautions”. Pairwise comparisons with adjusted^3^ p-values show that the difference between the youngest group and each of the two oldest groups are significant (*p*s < 0.003, *r*s > 0.24). All effect sizes are reported in the Appendix.

**Fig. 2:**
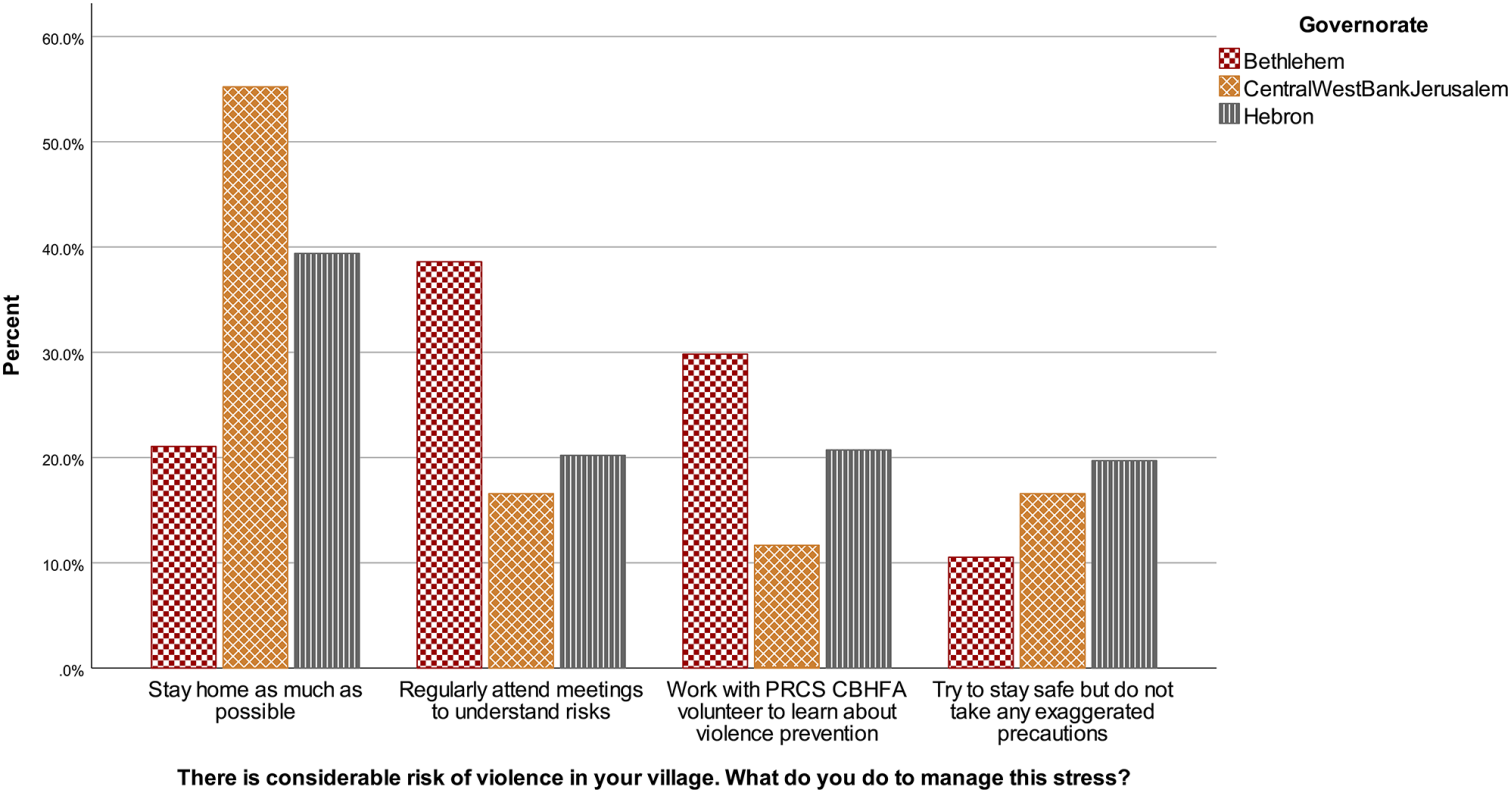
Violence situational judgement test answers according to governorate in the community sample, January-February 2023, Westbank region, occupied Palestinian territories Note. PRCS CBHFA = Palestine Red Crescent Society Community-Based Health and First Aid

There was no significant difference in answers according to gender, *U* = 20366.50, *z* = 0.083, *p* = .934. Answers varied according to governorate, *H* (2) = 11.84, *p* = .003. Figure 2 shows that answers from respondents from the Central governorate/West Bank/Jerusalem were more frequently “stay home”, compared to answers of respondents from Bethlehem and Hebron. Adjusted p-values indicate that these differences are significant (*p*s < 0.016, *r*s > − 0.15), but not Hebron compared to Bethlehem (*p* = .904).

**Fig. 3:**
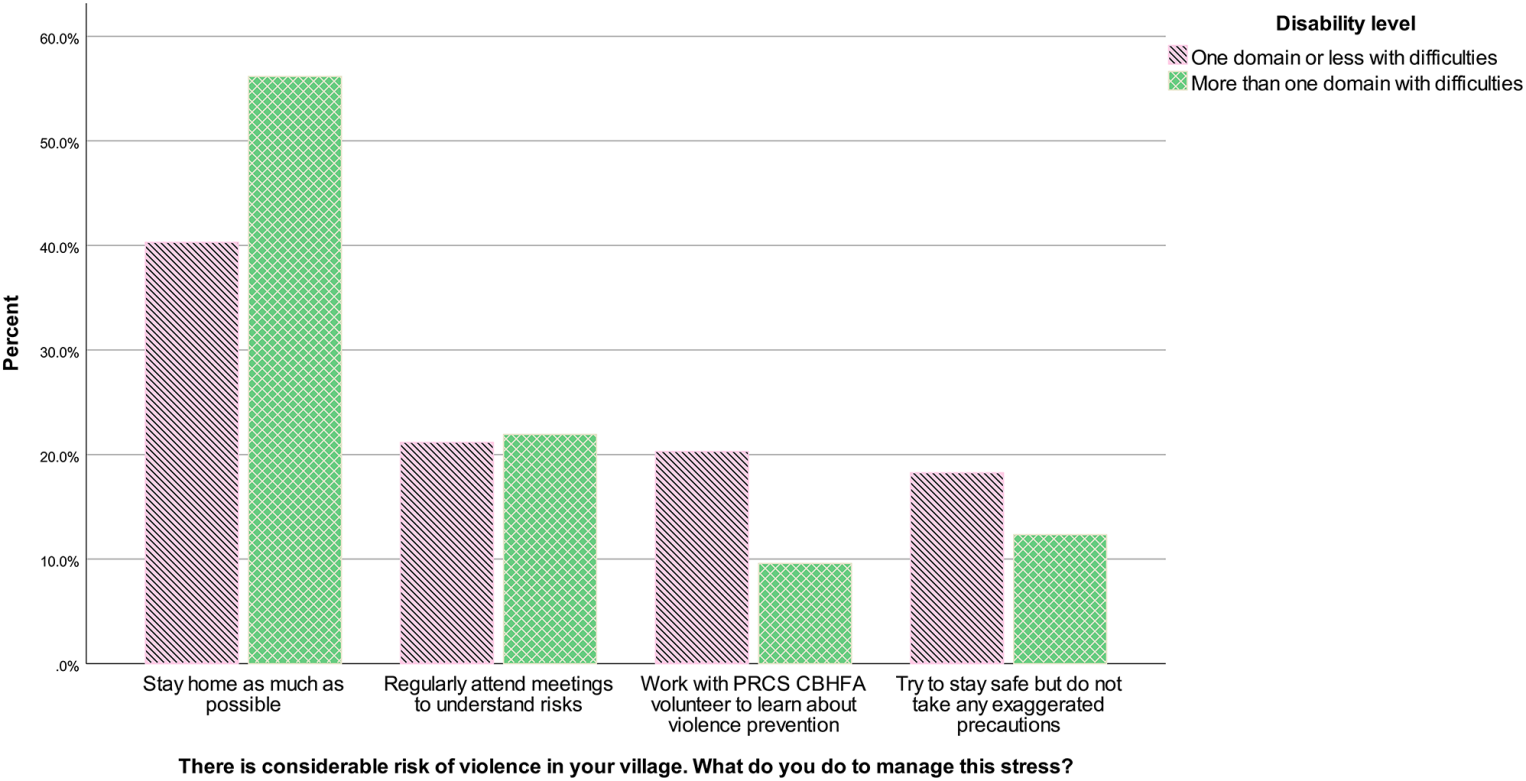
Violence situational judgement test answers according to disability level in the community sample, January-February 2023, Westbank region, occupied Palestinian territories *Note* PRCS CBHFA = Palestine Red Crescent Society Community-Based Health and First Aid

Answers to the violence SJT also differed according to disability level, *U* = 10208.00, *z* = -2.68, *p* = .007, *r* = − .13. Figure 3 indicates that respondents with disabilities (defined as having more than one domain of the scale with difficulties) answered more frequently staying at home as much as possible to manage stress related to violence, compared to respondents with no disabilities (one domain or less with difficulties).

### Violence SJT – volunteers

In the volunteer sample answering the violence SJT (*N* = 166), 16.9% of respondents chose the option “Stay at home as much as possible”, 30.7% chose the answer “Regularly attend meetings to understand risks”, 42.2% answered “Work with PRCS CBHFA volunteer to learn about violence prevention”, and 10.2% “Try to stay safe but do not take any exaggerated precautions”.

There were no differences in answer patterns according to the age of the volunteers, *H* (3) = 5.83, *p* = .120, neither according to their gender, *U* = 2621.00, *z* = 1.17, *p* = .243, nor governorate, *H* (2) = 0.76, *p* = .686, and disability, *U* = 1151.50, *z* = -1.32, *p* = .188.

### Road SJT – community

In the community sample answering the road safety SJT (*N* = 625), 8.3% of respondents chose the option “Tell your sons that they should not drive like you do”, 24.3% answered “Forbid your sons from driving with their friends”, 6.4% “Allow them to drive the family car only when you are with them”, 25.8% “Work with your PRCS CBHFA volunteer to lead youth sessions about road safety”, and 35.2% “Teach them the correct practices” (the best answer according to our coding and PRCS inputs).

**Fig. 4:**
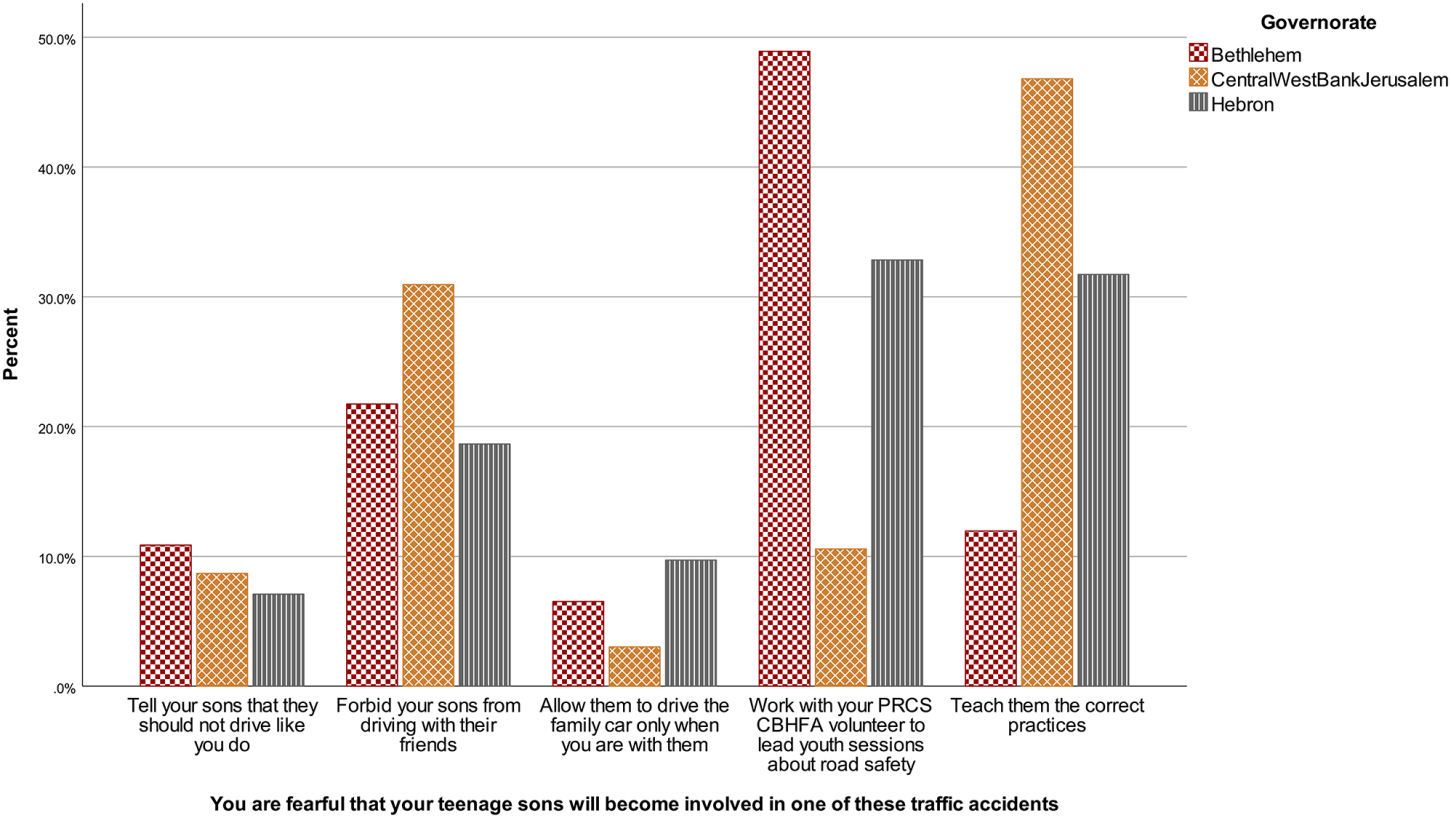
Road situational judgement test answers according to governorate in the community sample, January-February 2023, Westbank region, occupied Palestinian territories Note. PRCS CBHFA = Palestine Red Crescent Society Community-Based Health and First Aid

There were no differences in answer patterns according to the age of the respondents, *H* (3) = 2.84, *p* = .418, neither according to gender, *U* = 47515.50, *z* = 1.08, *p* = .280. Answers varied according to governorate, *H* (2) = 8.08, *p* = .018. Figure 4 shows that community members from Central/West Bank/Jerusalem answered more frequently to teach the correct practices to their children, while community members from Bethlehem answered more frequently to work with PRCS CBHFA volunteers. Adjusted p-values from pairwise comparisons show that Bethlehem answers differ from Hebron, *p* = .04, *r* = − .13, and Bethlehem also differ from Central/West Bank/Jerusalem, *p* = .016, *r* = − .15, but Hebron does not differ from Central/West Bank/Jerusalem, *p* = 1.00.

Answers to this SJT in the community did not vary according to disability level, *U* = 29963.00, *z* = -0.42, *p* = .675.

### Road SJT – volunteers

Among interviewed volunteers (*N* = 181), 3.9% chose the option “Tell them every day that they must be safe”, 10.5% answered “Forbid your sons from driving with their friends”, 29.3% “Allow them to drive the family car only when you are with them”, 39.8% “Work with your PRCS CBHFA volunteer to lead youth sessions about road safety”, and 16.6% “Teach them the correct practices”.

There were no differences in answer patterns according to the age of the volunteers, *H* (3) = 5. 24, *p* = .144, neither between male nor female respondents, *U* = 2558.50, *z* = -0.39, *p* = .697. Answers did not vary according to governorate, *H* (2) = 4.58, *p* = .101, nor disability level, *U* = 1236.50, *z* = -1.78, *p* = .076.

### Waste SJT – community

In the community sample answering the waste SJT (*N* = 231), 14.3% of respondents chose the option “Simply stack up the rubbish until the garbage collectors do come”, 9.5% answered “Give small bags of trash to different household members to get rid of each week wherever they can”, 36.8% “Watch out for vermin in the rubbish stacked up”, 29.9% “Discuss with your PRCS CBHFA volunteer about possibilities to reduce the waste problem”, and 9.5% “Throw the compostable waste into a bin for your garden” (the best answer according to our coding and PRCS inputs).

**Fig. 5:**
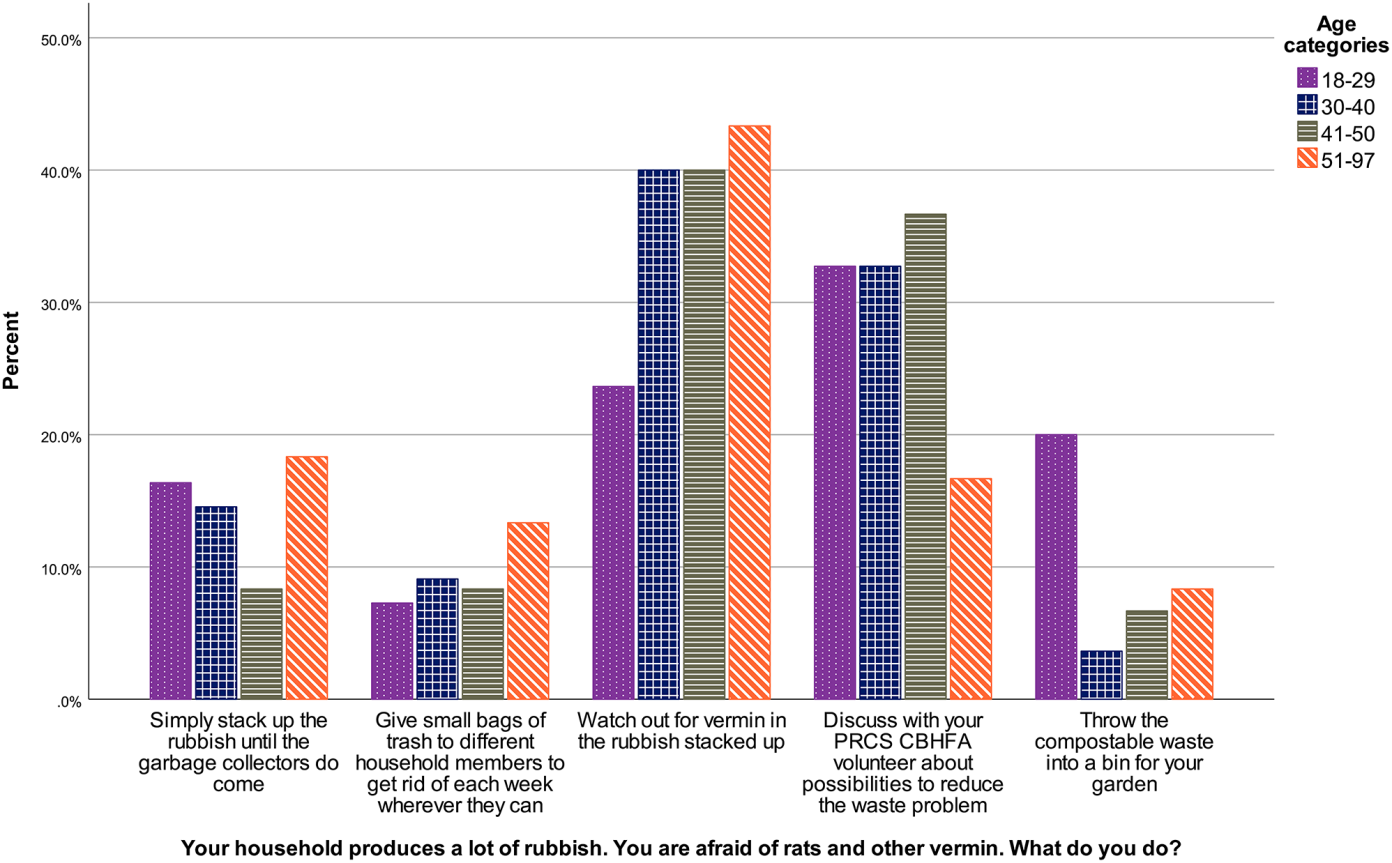
Waste situational judgement test answers according to age in the community sample, January-February 2023, Westbank region, occupied Palestinian territories Note. PRCS CBHFA = Palestine Red Crescent Society Community-Based Health and First Aid

Answers to this SJT varied according to the age of the respondent, *H* (3) = 8.10, *p* = .044. As visible in Fig. 5, the youngest group provides more often than older respondents the answer of doing home composting. Adjusted p-values of pairwise comparisons show that the comparison between the youngest and the oldest group misses the significance threshold (*p* = .051). All other comparisons are non-significant (*p*s > .233).

**Fig. 6:**
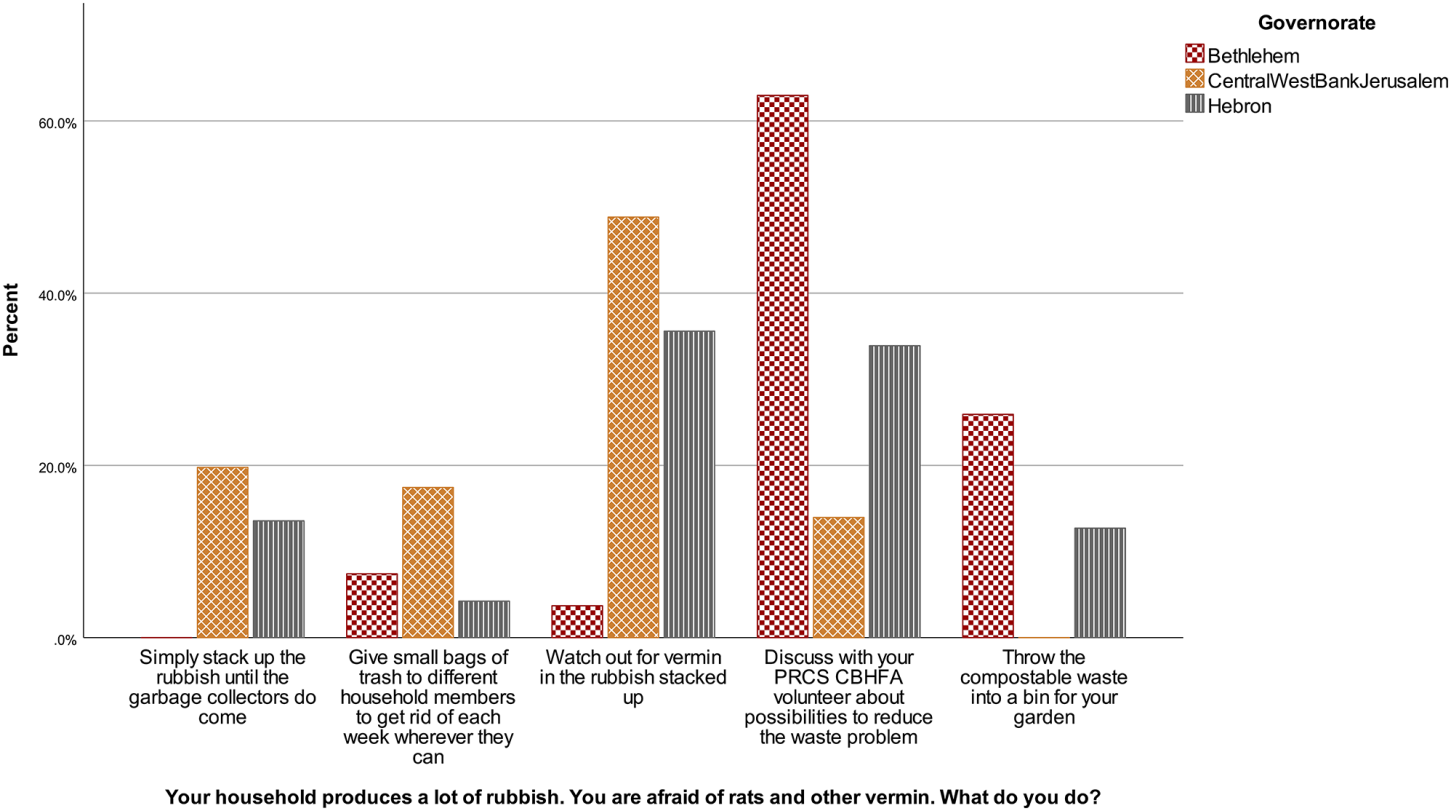
Waste situational judgement test answers according to governorate in the community sample, January-February 2023, Westbank region, occupied Palestinian territories Note. PRCS CBHFA = Palestine Red Crescent Society Community-Based Health and First Aid

No significant difference was observed between gender, *U* = 5918.00, *z* = -0.55, *p* = .585. However, significant difference was found across governorates, *H* (2) = 47.74, *p* < .001. Figure 6 presents the pattern of response. It is visible that respondents from Bethlehem are the ones most frequently answering “discuss with PRCS CBHFA volunteer”, while respondents from Central governorate provide answers on the lower end of the scale. All comparisons are statistically significant, Central/West Bank/Jerusalem vs. Hebron, *p* < .001, *r* = − .33; Central/West Bank/Jerusalem vs. Bethlehem, *p* < .001, *r* = .61; Hebron vs. Bethlehem, *p* = .001, *r* = .29.

No difference emerged for this SJT according to disability level, *U* = 3649.00, *z* = 0.39, *p* = .69.

### Waste SJT – volunteers

In the volunteers sample answering the waste SJT (*N* = 117), 8.5% of respondents chose the option “Simply stack up the rubbish until the garbage collectors do come”, 12.8% answered “Give small bags of trash to different household members to get rid of each week wherever they can”, 27.4% “Watch out for vermin in the rubbish stacked up”, 46.2% “Discuss with your PRCS CBHFA volunteer about possibilities to reduce the waste problem”, and 5.1% “Throw the compostable waste into a bin for your garden”.

**Fig. 7:**
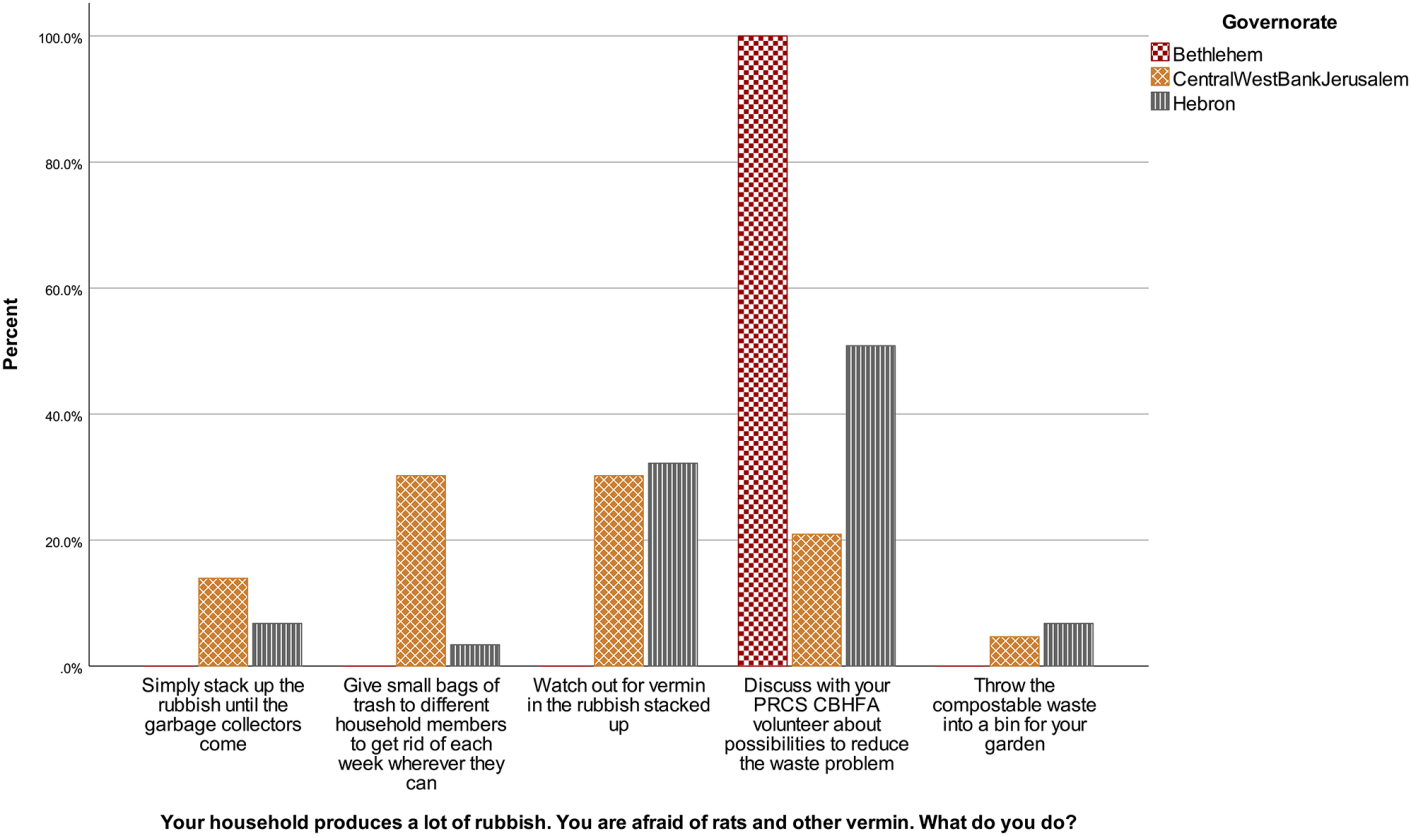
Waste situational judgement test answers according to governorate in the volunteer sample, January-February 2023, Westbank region, occupied Palestinian territories Note. PRCS CBHFA = Palestine Red Crescent Society Community-Based Health and First Aid

There were no differences in answer patterns according to the age of the volunteers, *H* (3) = 6.30, *p* = .098, neither between male and female respondents, *U* = 1043.50, *z* = -1.18, *p* = .237. Answers did vary according to governorate, *H* (2) = 24.44, *p* < .001. Results are presented in Fig. 7. The two comparisons that are statistically significant are Central vs. Bethlehem, *p* < .001, *r* = .59, and Central vs. Hebron, *p* = .001, *r* = − .36. The comparison between Hebron and Bethlehem is not significant, *p* = .104. No difference emerged according to disability level, *U* = 597.50, *z* = 0.14, *p* = .885.

### Sensitivity analysis- community

Because the response option mentioning PRCS could have led to more social desirability than the other ones, we conducted sensitivity analysis without this response option to test if differences emerged when testing patterns according to socio-demographic variables (age, gender), governorate and disability level.

No difference in what was statistically significant or not emerged for the road SJT analysis on the community sample.

For the waste SJT in the community sample, the age difference that was significant in the main analysis became non-significant when removing the option mentioning PRCS, *H* (3) = 2.49, *p* = .478. The other effects did not change (i.e., gender and disability level’s effects remained non-significant, and differences across governorate remained significant).

For the violence SJT in the community sample, all effects remained the same (i.e., age and governorate remained significant and gender remained non-significant) in the sensitivity analysis except for disability level. The disability level effect became non-significant (although it remained close from the significance threshold) in the sensitivity analysis, *U* = 7849.00, *z* = -1.88, *p* = .061.

### Sensitivity analysis- volunteers

**Fig. 8:**
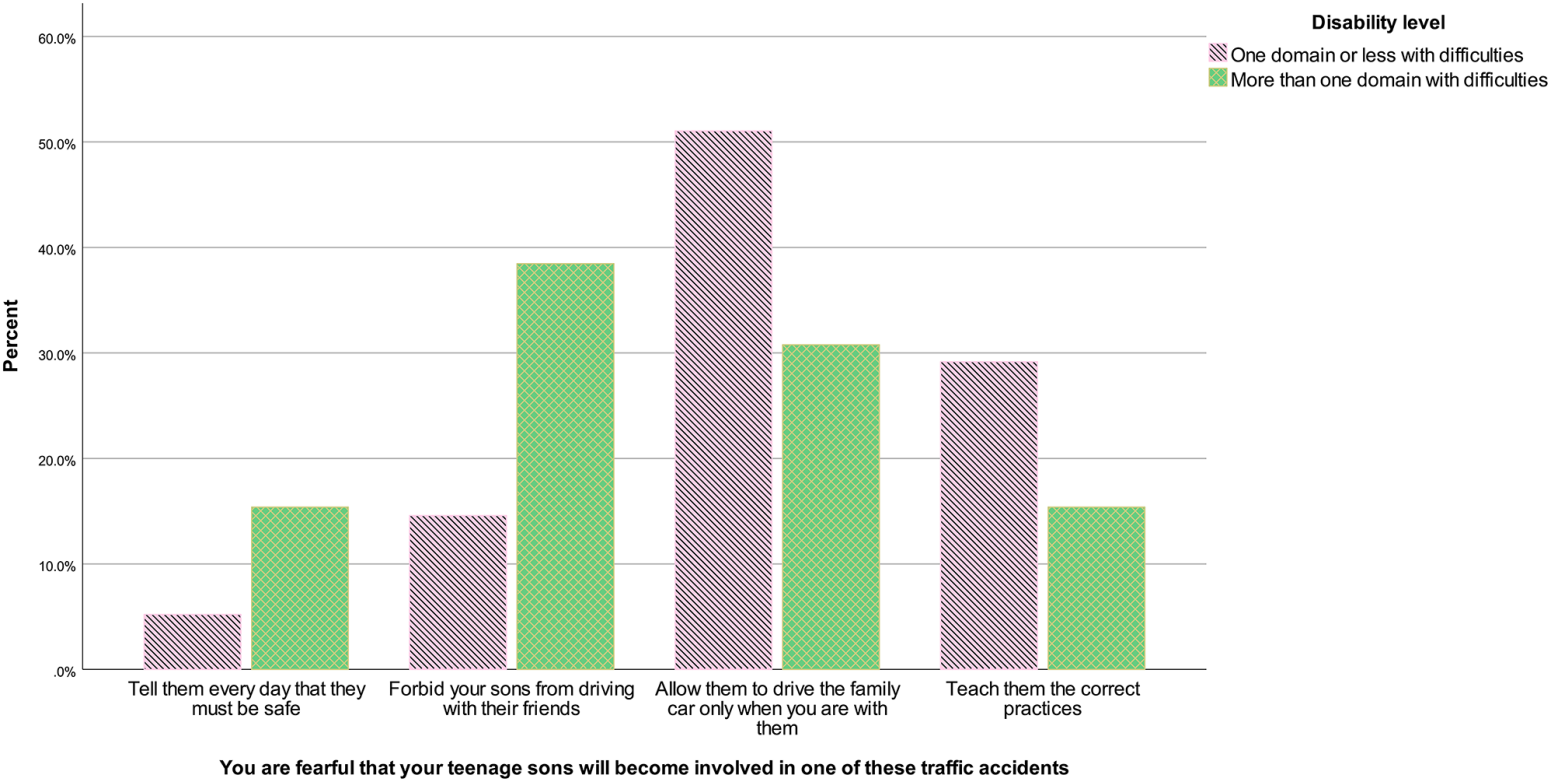
Road situational judgement test answers according to disability in the volunteers sample – sensitivity analysis, January-February 2023, Westbank region, occupied Palestinian territories

For the road SJT, no difference emerged in sensitivity analysis for age, gender or governorate. However, the effect of disability became significant, *U* = 403.00, *z* = -2.23, *p* = .026, *r* = − .21. As presented in Fig. 8, respondents with more than one domain with difficulties were more likely to answer to forbid their sons from driving with friends and tell them every day that they must be safe, and less likely to allow them to drive only when being with them and teach them the correct practices.

For the waste SJT, all effects remained similar to the main analysis (i.e., age, gender and disability remained non-significant, and governorate remained significant). Thus, there is no change in conclusion with sensitivity analysis for this SJT.

**Fig. 9:**
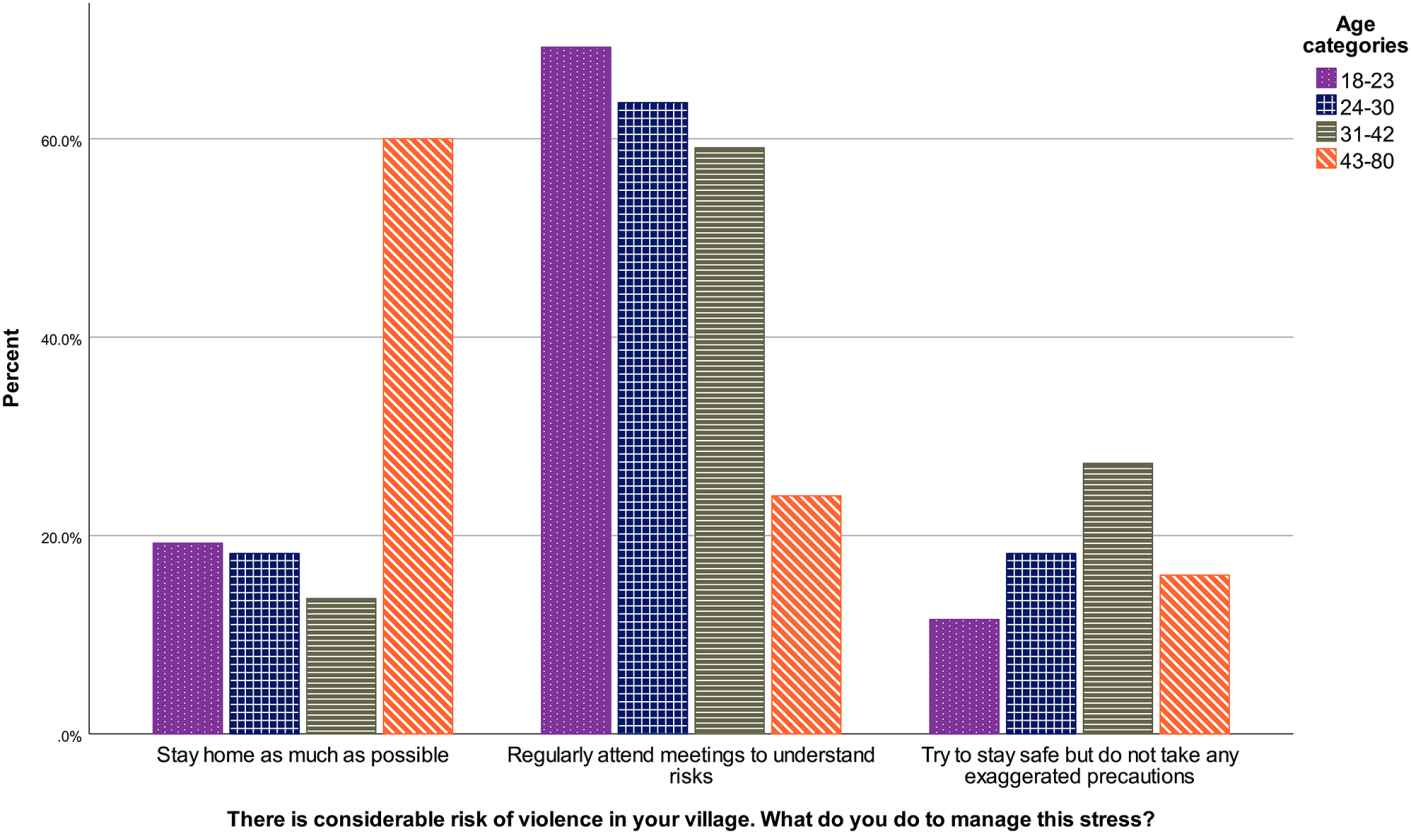
Violence situational judgement test answers according to age in the volunteers sample – sensitivity analysis, January-February 2023, Westbank region, occupied Palestinian territories

For the violence SJT, the age effect becomes significant when removing the category mentioning PRCS, *H* (3) = 10.28, *p* = .016. The adjusted pairwise comparisons show that it is the age groups of 31–42 and 43–80 that are significantly different from one another, as presented in Fig. 9. The older age group is more likely to answer staying home as much as possible, while the younger are more likely to attend meetings and try to stay safe without taking any exaggerated precautions.

**Fig. 10:**
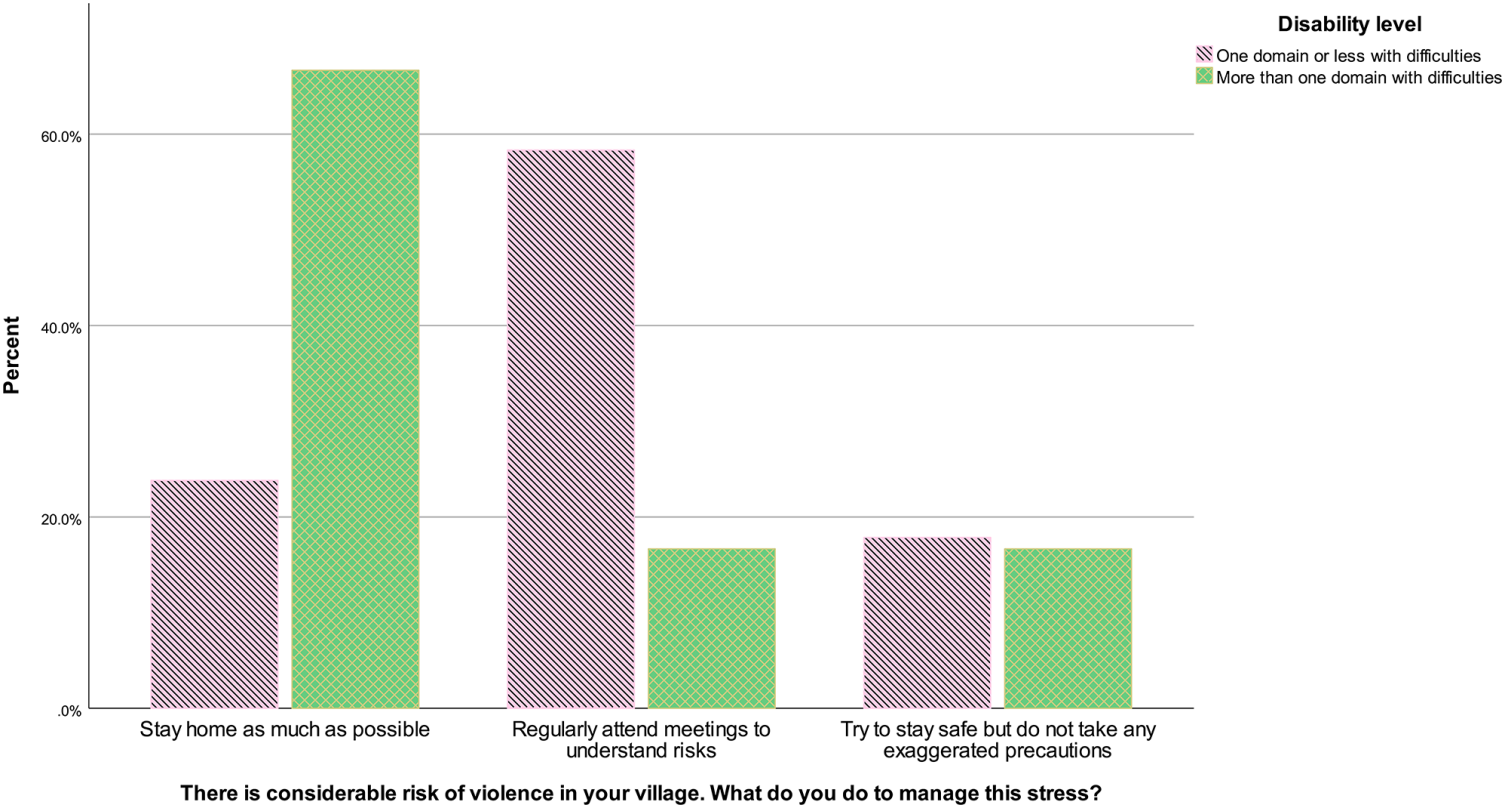
Violence situational judgement test answers according to disability in the volunteers sample – sensitivity analysis, January-February 2023, Westbank region, occupied Palestinian territories

Gender effect remains non-significant, and this was also the case for governorate’s effect. Disability effect becomes significant in the sensitivity analysis, *U* = 322.00, *z* = -2.23, *p* = .026, *r* = − .23. Figure 10 shows that volunteers with more than one domain with difficulties are more likely to answer staying home as much as possible, and less likely to attend meetings to understand risks.

## Discussion

This paper reports result of a field study using SJTs to collect community insights towards three priority topics, violence prevention, road safety, and environmental pollution (waste), in selected West Bank Palestinian communities and Red Crescent volunteers. Despite the limitations described below, the study shows that the tool worked to measure norms and beliefs in a standardized manner. An important result is that response patterns sometimes vary according to age, governorate, or disability level. This shows that the tool was able to detect those differences. It also highlights the need to target subgroups differently concerning those topics. Perhaps surprisingly, no significant difference emerged according to the gender of the respondents, neither in the community nor in the volunteer sample. This lack of difference seems to suggest that both genders share norms on those three topics.

### Implications for specific SJT - violence prevention

We see that most community members – especially those with more than one disability – as well as older volunteers choose to stay home in times of violence. PRCS’ household visits can be supplemented with online WhatsApp support groups and other ways to reach people in their homes where they feel safest.

The best answer to the SJT question was to stay safe but to not take exaggerated precautions, to trust your instinct, but only 10.2% of volunteers chose this response.

Most volunteers chose the support of PRCS and attending meetings to stay aware of risks. These responses show the cultural norm of tightly formed networks of support in the communities and the reliance on PRCS and community meetings to plan. PRCS volunteers seek safety and reassurance amongst PRCS volunteers more than self-reliance. This shows the Palestinian norm of its strength in working in community and can be fostered. There can be sessions to coach in leadership skills.

### Implications for specific SJT – road safety

The best answer to the SJT question was the most frequent response for community members in teaching sons to drive properly. The volunteers’ most frequent response was to work with the PRCS. This highlights how volunteers uphold PRCS as a part of their support base, with many volunteers wanting more training on road safety. Meanwhile community members want to model behaviours – one of the most effective ways of changing behaviours. This suggests that volunteers would benefit from some form of training but also practical exercises to encourage road safety practices. Using community members as models of good driving would be incredibly valuable to both volunteers and community.

### Implications for specific SJT – environmental pollution (waste)

The best answer, ‘To throw compost in a bin to use in the garden to provide nutrients to plants’ was only chosen by 9.5% of the community respondents and 5.1% of volunteers. Community members are more aware of sustainable environmental pollution solutions than some of their volunteers. Yet much more education and awareness are needed. Activities can focus on ways to reduce waste that also encourages household gardens. The most popular responses for community were to watch out for vermin in the trash, and talking to the PRCS volunteer or branch supervisor for volunteers (and the second most frequent option for community members). This indicates a high level of expectation of PRCS supervisors and volunteers. Training staff and volunteers on sustainable waste management strategies like composting and household gardens is strongly suggested. There is also a chance to teach about what to do to keep safe when vermin are spotted.

### Limitations

One limitation of our study is that the number of volunteers surveyed is smaller than the number of community members, leading to smaller probabilities of finding, if they exist, significant effects of demographic variables or geographical differences. Additionally, we used the same volunteers as enumerators for both the volunteer survey and the community survey. This was done for safety reasons and to ensure greater response as the PRCS is a trusted entity in communities where risk is high and outside enumerators may not have been effective. However, the downside is that volunteers interviewing other volunteers might have triggered a motivation to give “good” answers. As far as possible, future studies should try to rely on external enumerators for the volunteers group.

A limitation of the use of SJTs or other tools (e.g., Knowledge Attitudes Practices surveys) is the time and lack of fully-qualified staff to conduct the assessment and analyze the data, as highlighted by respondents in the study by White et al. [39]. Aside from the question of whether the resources to conduct data collection were available, a more complex issue is the fact that resources used for data collection could have been used differently, for example in funding relief and/or aid to those in need [12]. We argue data collected must be later used to inform programs or monitor existing actions to be able to consider that resources were used efficiently. Regarding the question of limited resources and staff, making developed SJTs publicly available as we do here ((see also 19)) may help other teams to build from existing resources and save time in tool development.

We want to point out that the hierarchy in response options can be challenged and could have been done otherwise. Our hierarchy was discussed with the various team members, and we followed the opinion of local members in the perspective that they know best what works for them in their context. For example, in the violence prevention SJT, the options to do nothing special and trust your intuition to stay safe could be perceived as insufficient from an external point of view, but was considered best by Palestinian members of the team because otherwise, people would be spending all their times in meetings due to the long-standing and pervasive issue of violence.

A number of difficulties arose during data collection, and are worth mentioning to put in perspective our results. These include: (A) four iterations of the survey were deployed during the planned data collection period due to mistakes in implementing the survey on the data collection platform. We eliminated all data that was incomplete, suspect or unreliable, which resulted in a loss of many data points. (B) ODK and the server on which it runs was not functioning on the day of the enumerator training which limited our ability to demonstrate and allow for practice of the survey in a training environment. (C) The printed survey used in the enumerator training did not reflect the most updated survey – thus the enumerator training was minimally helpful to ensuring that enumerators followed the sampling strategy or even conducted the survey effectively. (D) We were unable to adhere to sampling strategy, because there were two significant security incidents during the data collection period. It was deemed unsafe for enumerators to collect data face-to-face in some areas according to the sampling strategy. It was agreed that enumerators could call people within the collection area to administer the survey and apply snowball sampling for additional potential respondents. This diversion in strategy meant that the data was not collected from a random selected sample. Because the sample is not randomly selected, respondents might not be representative of the general population, thus limiting generalizations that can be drawn from the survey data. For example, it is possible that PRCS volunteers have socio-economic characteristics different from those of the general population (in terms of literacy and education level, for example), and people recruited via snowball sampling might share the same characteristics, creating a sampling bias [40]. (E) While the capacity of volunteers to serve as enumerators is helpful, it does lend bias when volunteers are asking community members questions around PRCS work. This is the reason why we conducted the sensitivity analysis without the response option mentioning PRCS. The latter two limitations deserve more elaboration in the specific context of oPt, which we will delve into below.

These limitations may be of strong concern to readers unfamiliar with the day-to-day complex context of life in occupied Palestinian territories. But for those who work and serve the people within these communities, we argue that despite the challenges, the need for reliable data sets is a worthwhile and necessary endeavour. The data captured in one of the most complex environments is important to ensuring that interventions and projects respond to the ongoing challenges faced by community members across occupied Palestine. The oPt is a unique context where Palestinians often suffer from a significant imbalance of power. There are imposed limits to who can access parts of oPt – including Palestinians within oPt, thus making it very difficult for outside evaluators or even Palestinians to deploy as enumerators that are unknown to the communities. With random household sampling, this would include having unknown persons visiting respondents’ homes and schools. In this context, this was not feasible or ethical. Further, the PRCS is an organisation that almost all Palestinians are familiar with – many relying on their health units, clinics and volunteers who are a ready and constant presence, in peace and in conflict, often providing assistance when there are no other organisations operating or assisting. The decision was made with PRCS to deploy community volunteers within their own communities to collect data to ensure that people felt comfortable and could answer without anxiety. Volunteers are a trusted source of information and all have vests and ID cards to identify their affiliation with PRCS. Volunteers at PRCS are familiar with the data collection tool and most already had it installed on their mobile phones.

We trained the volunteer enumerators in random sampling. However, a violent attack on Palestinians occurred in the West Bank the day that data collection was to begin. The communities’ collective anxiety greatly contributes to Palestinians’ collective apprehension of people outside of their community and this incident posed the same fear. In speaking with local volunteers after the incident, the team was informed that people were not answering their doors to those they did not know – thus greatly affecting our plan to conduct random sampling. The volunteers said that in communicating with other volunteers, the only way that data collection would occur at our anticipated scale and in the environment of fear and intimidation, is through the snowball method. We had a call with the programme lead regarding how to discuss the shift to non-random sampling. We prepared guidance to communicate to volunteers on how to use the snowball method to reduce bias. The programme lead explained that the selected staff and volunteers were well-versed in the method due to regular occurrences of violence against the population and the need to shift methods. When asked if a follow-up training and support were needed, we were informed that due to the halt on movement within select areas of the West Bank, a follow-up face-to-face training was not possible. The programme lead sent texts to Programme Coordinators about employing the snowball method who, in turn, shared with the volunteers.

Future studies’ perspectives include increasing data quality, for example, using triangulation of multiple methods [41] to avoid relying on only one data source whose feasibility could be jeopardized due to external circumstances. Another direction could be mobile technology, which could influence accessibility [42], if enumerators no longer need to interview respondents face-to-face. However, the feasibility of using such methods will depend on the available resources and the local context. As Axinn et al. concluded in their paper [43], data collection during armed conflict required tailoring to adapt to the circumstances.

## Conclusions

This study shows that SJTs can be used to measure communities’ beliefs and norms about various topics, ranging from environmental pollution to violence and road safety. We hope that this step in developing a tool to capture local needs and priorities will help increase community engagement and ensure genuine local leadership.

### Electronic supplementary material

Below is the link to the electronic supplementary material.

Supplementary Material 1

## Data Availability

The datasets generated and analysed during the current study are available in the OSF repository, https://osf.io/p98s6/?view_only=fc0945d2ecfb40bbb64fff550b604fc6.

## Footnotes

2 N varies from the initial sample size due to missing data.

3 significance values adjusted by the Bonferroni correction to take into account multiple testing.

## Abbreviations

SJT: Situational Judgement Tests
CBHFA: Community-Based Health and First Aid
PRCS: Palestine Red Crescent Society
oPt: Occupied Palestinian territories
