# Supplementary material for: Situational Judgement Tests among Palestinian community members and Red Crescent volunteers to inform humanitarian action: a cross-sectional study
Source: Arch Public Health. 2024 Aug 27;82:141. doi: 10.1186/s13690-024-01356-8 (PMC11351291; doi:10.1186/s13690-024-01356-8)
Supplement: Supplementary file 1 — Supplementary Material 1 [file 13690_2024_1356_MOESM1_ESM.docx]

Appendix

Supplementary Table 1. Effect sizes of the statistical comparison. Study: Situational judgement tests. January-February 2023, Westbank region, occupied Palestinian territories

| **sample** | **SJT** | **Categorical variable** | **comparison** | **standardized test statistic** | **N** | **Effect size r** |
| --- | --- | --- | --- | --- | --- | --- |
| community | violence | age | 55-45 | 1,338 | 210 | 0,092 |
|  |  |  | 55-35 | 2,329 | 204 | 0,163 |
|  |  |  | 55-25 | 4,717 | 202 | 0,332 |
|  |  |  | 45-35 | 1,033 | 214 | 0,071 |
|  |  |  | 45-25 | 3,484 | 212 | 0,239 |
|  |  |  | 35-25 | 2,423 | 206 | 0,169 |
|  |  | gender | H-F | 0,083 | 418 | 0,004 |
|  |  | governorate | central/hebron | -2,784 | 361 | -0,147 |
|  |  |  | central/bethlehem | 2,923 | 220 | 0,197 |
|  |  |  | hebron/bethlehem | 1,033 | 255 | 0,065 |
|  |  | disability | one domain or less vs. more than one | -2,68 | 418 | -0,131 |
|  | road safety | age | 55-45 | 0,233 | 306 | 0,013 |
|  |  |  | 55-35 | 0,056 | 307 | 0,003 |
|  |  |  | 25-55 | -1,236 | 298 | -0,072 |
|  |  |  | 35-45 | -0,183 | 325 | -0,010 |
|  |  |  | 25-45 | -1,51 | 316 | -0,085 |
|  |  |  | 25-35 | -1,332 | 317 | -0,075 |
|  |  | gender | H-F | 1,081 | 625 | 0,043 |
|  |  | governorate | bethlehem/hebron | -2,47 | 360 | -0,130 |
|  |  |  | bethlehem/central | -2,78 | 357 | -0,147 |
|  |  |  | hebron/central | 0,435 | 533 | 0,019 |
|  |  | disability | one domain or less vs. more than one | -0,419 | 625 | -0,017 |
|  | waste | age | 55-35 | 1,001 | 115 | 0,093 |
|  |  |  | 55-45 | 2,065 | 120 | 0,189 |
|  |  |  | 55-25 | 2,628 | 115 | 0,245 |
|  |  |  | 35-45 | -1,019 | 115 | -0,095 |
|  |  |  | 35-25 | 1,593 | 110 | 0,152 |
|  |  |  | 45-25 | 0,609 | 115 | 0,057 |
|  |  | gender | H-F | -0,547 | 231 | -0,036 |
|  |  | governorate | central/bethlehem | 6,43 | 113 | 0,605 |
|  |  |  | central/hebron | -4,74 | 204 | -0,332 |
|  |  |  | hebron/bethlehem | 3,5 | 145 | 0,291 |
|  |  | disability | one domain or less vs. more than one | 0,394 | 231 | 0,026 |
| volunteers | violence | age | 45-20 | 1,199 | 83 | 0,132 |
|  |  |  | 45-25 | 2,124 | 84 | 0,232 |
|  |  |  | 45-35 | 2,039 | 74 | 0,237 |
|  |  |  | 20-25 | -0,961 | 91 | -0,101 |
|  |  |  | 20-35 | -0,94 | 81 | -0,104 |
|  |  |  | 25-35 | -0,039 | 82 | -0,004 |
|  |  | gender | H-F | 1,167 | 166 | 0,091 |
|  |  | governorate | central/hebron | -0,584 | 135 | -0,050 |
|  |  |  | bethlehem/central | -0,335 | 93 | -0,035 |
|  |  |  | bethlehem/hebron | -0,814 | 104 | -0,080 |
|  |  | disability | one domain or less vs. more than one | -1,317 | 166 | -0,102 |
|  | road | age | 20-45 | -0,538 | 90 | -0,057 |
|  |  |  | 20-35 | -1,32 | 91 | -0,138 |
|  |  |  | 20-25 | -2,19 | 94 | -0,226 |
|  |  |  | 45-35 | 0,763 | 87 | 0,082 |
|  |  |  | 45-25 | 1,603 | 90 | 0,169 |
|  |  |  | 35-25 | 0,833 | 91 | 0,087 |
|  |  | gender | H-F | -0,39 | 181 | -0,029 |
|  |  | governorate | bethlehem/hebron | -1,215 | 99 | -0,122 |
|  |  |  | bethlehem/central | -2,106 | 113 | -0,198 |
|  |  |  | hebron/central | 1,101 | 150 | 0,090 |
|  |  | disability | one domain or less vs. more than one | -1,1775 | 181 | -0,088 |
|  | waste | age | 45-35 | 0,974 | 52 | 0,135 |
|  |  |  | 45-25 | 1,764 | 57 | 0,234 |
|  |  |  | 45-20 | 2,382 | 55 | 0,321 |
|  |  |  | 35-25 | 0,787 | 61 | 0,101 |
|  |  |  | 35-20 | 1,444 | 59 | 0,188 |
|  |  |  | 25-20 | 0,697 | 64 | 0,087 |
|  |  | gender | H-F | -1,184 | 117 | -0,109 |
|  |  | governorate | central/bethlehem | 4,48 | 58 | 0,588 |
|  |  |  | central/hebron | -3,66 | 102 | -0,362 |
|  |  |  | hebron/bethlehem | 2,113 | 74 | 0,246 |
|  |  | disability | one domain or less vs. more than one | 0,144 | 117 | 0,013 |
